# Medical College of Missouri

**Published:** 1842-09-24

**Authors:** 


*Medical College of Missouri.* The faculty of this institution is now organised as follows:—Daniel Brainard, M. D., Professor of Anatomy and Physiology; Josephus W. Hall, M. D., Professor of Theory and Practice of Medicine and Clinical Practice; H. Augustus Prout, M. D., Professor of Chemistry, Materia Medica and Medical Botany; James V. Prather, M. D., Professor of Surgery and of Surgical and Pathological Anatomy; Moses L. Linton, M. D., Professor of Obstetrics and Diseases of Women and Children.

